# Lack of TRPM5-Expressing Microvillous Cells in Mouse Main Olfactory Epithelium Leads to Impaired Odor-Evoked Responses and Olfactory-Guided Behavior in a Challenging Chemical Environment

**DOI:** 10.1523/ENEURO.0135-17.2017

**Published:** 2017-06-12

**Authors:** Kayla Lemons, Ziying Fu, Imad Aoudé, Tatsuya Ogura, Julianna Sun, Justin Chang, Kenechukwu Mbonu, Ichiro Matsumoto, Hiroyuki Arakawa, Weihong Lin

**Affiliations:** 1 Department of Biological Sciences, University of Maryland, Baltimore County, Baltimore, MD; 2 Monell Chemical Senses Center, Philadelphia, PA; 3 Department of Research Administration, School of Medicine, Case Western Reserve University, Cleveland, OH

**Keywords:** Olfactory behaviors, olfactory epithelium, olfactory responses, TRPM5, TRPM5-expressing microvillous cells

## Abstract

The mammalian main olfactory epithelium (MOE) modifies its activities in response to changes in the chemical environment. This process is essential for maintaining the functions of the olfactory system and the upper airway. However, mechanisms involved in this functional maintenance, especially those occurring via paracrine regulatory pathways within the multicellular MOE, are poorly understood. Previously, a population of non-neuronal, transient receptor potential M5-expressing microvillous cells (TRPM5-MCs) was identified in the MOE, and the initial characterization of these cells showed that they are cholinergic and responsive to various xenobiotics including odorants at high concentrations. Here, we investigated the role of TRPM5-MCs in maintaining olfactory function using transcription factor Skn-1a knockout (Skn-1a^-/-^) mice, which lack TRPM5-MCs in the MOE. Under our standard housing conditions, Skn-1a^-/-^ mice do not differ significantly from control mice in odor-evoked electro-olfactogram (EOG) responses and olfactory-guided behaviors, including finding buried food and preference reactions to socially and sexually relevant odors. However, after a 2-wk exposure to high-concentration odor chemicals and chitin powder, Skn-1a^-/-^ mice exhibited a significant reduction in their odor and pheromone-evoked EOG responses. Consequently, their olfactory-guided behaviors were impaired compared with vehicle-exposed Skn-1a^-/-^ mice. Conversely, the chemical exposure did not induce significant changes in the EOG responses and olfactory behaviors of control mice. Therefore, our physiological and behavioral results indicate that TRPM5-MCs play a protective role in maintaining the olfactory function of the MOE.

## Visual Abstract

**Figure:**
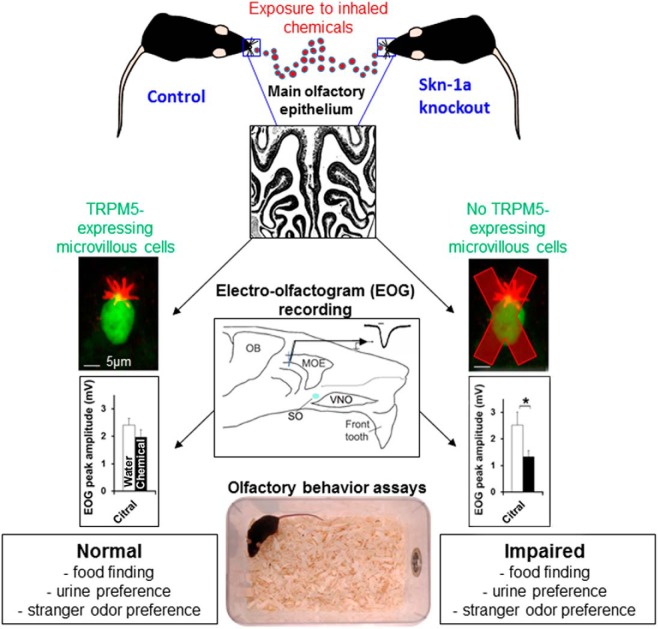

## Significance Statement

The main olfactory epithelium (MOE) detects odor molecules and provides sensory inputs for behavioral guidance and modification of the psychological state. Additionally, the MOE protects the brain and respiratory organs by providing an epithelial barrier and biotransforming xenobiotics. The MOE directly faces the respiratory airstream and is vulnerable to damage caused by inhaled odorous irritants, pollutants, and infectious agents. Little is known about the mechanisms that detect xenobiotics and modulate MOE activity for functional maintenance. This study shows that transient receptor potential M5-expressing microvillous cells (TRPM5-MCs) play an important role in maintaining MOE physiological responses to odorants and pheromones in a challenging chemical environment, and subsequently olfactory-guided behaviors. Therefore, these results revealed a novel TRPM5-MC–mediated intercellular regulatory pathway.

## Introduction

The mammalian MOE serves three distinct functions. First, it detects thousands of odor molecules and provides sensory inputs that critically influence the brain’s psychological state and guide food foraging, habitat selection, mate choice, and social interaction (Buck, 2004; Restrepo et al., 2004; Tirindelli et al., 2009). Second, the MOE serves as an epithelial surface barrier, preventing or minimizing inhaled xenobiotics, such as air pollutants and infectious agents, from entering the brain, where they can create inflammation and neurodegeneration (Prediger et al., 2006; Imamura and Hasegawa-Ishii, 2016). Third, the MOE serves as a primary site for metabolizing and removing xenobiotics in the upper respiratory tract (Chen et al., 1992; Thornton-Manning et al., 1997; Thiebaud et al., 2010). The MOE is susceptible to xenobiotic insults owing to its large surface area and direct contact with inhaled chemicals. However, little is known about mechanisms that detect xenobiotics and subsequently modulate olfactory activity for functional maintenance.

The MOE is made up of olfactory sensory neurons (OSNs), supporting cells (SCs), basal cells, microvillous cells (MCs), and the cells of Bowman’s glands and ducts (Morrison and Costanzo, 1992; Farbman, 2000). The MCs display diverse morphology in their apical microvilli and basal processes (Menco and Morrison, 2003), with distinct molecular features. One MC population exhibits short and cone-shaped microvilli. These cells express transient receptor potential channel C6 (TRPC6), respond to odor molecules, and promote MOE adult neurogenesis (Elsaesser and Paysan, 2007; Hegg et al., 2010; Jia et al., 2013). Another population of MCs expresses TRPM5 (Lin et al., 2007, 2008b; Hansen and Finger, 2008). These TRPM5-MCs reside superficially throughout the MOE, with their apical microvilli extending into the mucus layer (Lin et al., 2008b; Yamaguchi et al., 2014). They differ from TRPC6-MCs in that TRPM5-MCs exhibit elaborate apical microvilli and generally lack a basal process connecting them to the basal lamina. Also, TRPM5-MCs differ from OSNs in that they do not express neuronal or OSN markers and components of the canonical olfactory transduction pathway (Lin et al., 2008b). Therefore, TRPM5-MCs represent a distinct population in the MOE.

Interestingly, TRPM5-MCs express the cholinergic markers choline acetyltransferase (ChAT) and vesicular acetylcholine transporter (VAChT). Also, TRPM5-MCs respond to various chemical stimuli, such as odorous chemicals, ATP, and bacterial lysate, with increases in intracellular Ca^2+^ levels (Ogura et al., 2011). These findings led the investigators to hypothesize that TRPM5-MCs detect harmful chemicals and subsequently regulate MOE activities for functional maintenance. Currently, TRPM5-expressing chemosensory cells capable of detecting various harmful chemicals have been found in a variety of tissues (Finger et al., 2003; Bezencon et al., 2008; Gulbransen et al., 2008; Lin et al., 2008a; Ogura et al., 2010; Krasteva et al., 2011; Tizzano et al., 2011; Deckmann et al., 2014). However, it is not known how TRPM5-MCs contribute to the overall function of the MOE.

This study sought to determine the physiological and behavioral role of TRPM5-MCs in maintaining proper MOE functions. Knockout of the POU homeobox transcription factor Skn-1a (Skn-1a^-/-^) eliminates TRPM5-expressing chemosensory cells, including TRPM5-MCs, in the MOE (Matsumoto et al., 2011; Yamaguchi et al., 2014). We first determined whether the lack of TRPM5-MCs would affect evoked electro-olfactogram (EOG) responses to odorants and pheromones in mice housed under standard conditions. We then determined whether TRPM5-MCs play a role in MOE maintenance by monitoring EOG responses and performing immunohistological examination after animals were exposed to a mixture of relatively high concentrations of odorants and substances commonly found in the environment and occupational settings. Furthermore, we assayed olfactory ability in guiding behaviors of food searching and preference toward sexually and socially relevant odors. Our data demonstrate that after 2-wk chemical exposure, there was significant impairment in EOG responses and olfactory-guided behaviors in Skn-1a^-/-^ mice but not in control mice, compared with their respective paired vehicle-exposed groups. Therefore, our results provide the first evidence that TRPM5-MCs play an important role in maintaining olfactory function and guided behavior.

## Materials and Methods

### Animals

Two- to six-month-old adult male and female C57BL/6 background transgenic and knockout mice were used in this study. For physiologic recordings, mice of both sexes were used. For behavioral experiments, only male mice were used. The TRPM5-GFP transgenic mouse line, in which the promoter of TRPM5 drives the expression of GFP, was originally generated in Robert R. Margolskee’s laboratory (Clapp et al., 2006). This mouse line has been used in previous studies of TRPM5-MCs (Lin et al., 2008b; Ogura et al., 2011). In this study, TRPM5-GFP mice were used as controls, and we refer to them as such throughout the article. The Skn-1a^-/-^ line of mice (RRID:IMSR_RBRC05254) was originally generated by Matsumoto et al. (2011). The lack of TRPM5-MCs in the MOE of these mice has been characterized previously (Yamaguchi et al., 2014). In standard housing conditions, mice were group-housed (two to five mice per cage) in an open-top cage system in our animal facility. Cages were changed weekly, and water and food were available *ad libitum*. All animal care and use procedures were conducted in accordance with the National Institutes of Health Guide for the Care and Use of Laboratory Animals (2006) and approved by the Animal Care and Use Committee of the University of Maryland, Baltimore County.

### Chemical stimuli and solutions

Odorants and chemicals for solutions were purchased from Sigma-Aldrich at the highest purity available. Stock solutions of odorants were made and stored in a –20°C freezer. Odorants for EOG recording were freshly made by dilution with vigorous vortexing into Ringer’s saline (for EOG recordings; in mm: 145 NaCl, 5 KCl, 10 HEPES, 1 MgCl_2_, 1 CaCl_2_, 1 Na pyruvate, and 5 d-glucose, pH 7.2). The adenylyl cyclase activator forskolin was purchased from Calbiochem. Fine chitin powder was purchased from TCI. For urine collection from group-housed adult C57BL/6 female mice, two methods were used. In the first method, individual mice were handheld, and gentle pressure was applied to the lower abdomen to facilitate urination. In the second method, individual mice were placed into a clean cage without bedding, and urine samples were collected after secretion. The urine samples from individual animals were pooled, aliquoted (200 µl each), quickly frozen, and stored at –80°C. Freshly thawed urine samples were used in experiments. Water samples for the vehicle-exposed control mice and the T-maze odor preference test were obtained from the same tap water source.

### Chronic exposure to chemical stimuli

Mice used for control and exposure conditions were matched for age and sex and group-housed in isolated filter-top cages (28 cm long by 19 cm wide by 12 cm high). Animals had free access to food and water throughout the duration of the experiment. The odor mixture for 2-wk exposure included ammonium hydroxide, ethyl acetate, propionic acid, and triethylamine, which were made up individually and mixed before use to a final concentration of 0.019, 0.075, 0.083, and 0.013 m, respectively. These chemicals were selected largely on the basis of their widespread use in various industrial applications, the possibility of them being inhaled by animals in natural conditions, and their relevance to occupational health/exposure toxicology (Kuwabara et al., 2007). The concentrations of these odors were chosen based on occupational health guidelines for irritation levels (www.cdc.gov/niosh/) and the RD_50_ value, which is the concentration producing a 50% respiration rate decrease (Kuwabara et al., 2007; van Thriel et al., 2008). The gas phase concentrations for daily exposure, which were derived by the vapor pressure of the mixture at 20–25°C, were estimated to be 52 ppm for ammonium hydroxide, 180 ppm for ethyl acetate, 4.8 ppm for propionic acid, and 17 ppm for triethylamine, assuming the cage was closed. The intention was to challenge, but not overwhelm, the MOE’s ability to maintain its integrity so that we could assess the effects of the exposure on Skn-1a^-/-^ mice.

For chemical exposure, a small glass vial (either 1 cm diameter by 6.5 cm height or 1.8 cm diameter by 7 cm height) containing 4 ml of the chemical mixture with a piece of Kimwipe was placed in the cage. The odor mixture was refilled daily, and mice were exposed either continuously for 14 d or on weekdays only for at least 14 d total. In addition to the exposure to odorous volatiles, mice in the chemical exposure group were also transferred to a new cage and exposed to chitin powder (250 mg/cage) for 10 min daily. Chitin is a characteristic component making up the cell walls of fungi and the exoskeletons of arthropods and insects. Chitin powder was chosen because it can be found abundantly in nature. A small air pump was used to blow air around the cage to prevent the powder from accumulating in the corners or being moistened by urine. Water control groups from both mouse lines were exposed only to vehicle (water).

### EOG recordings

**Figure 1.**
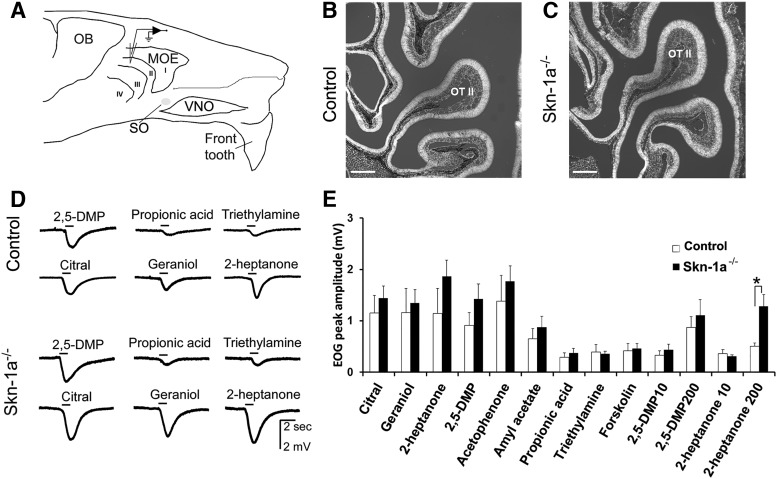
Odorants and pheromones evoke comparable EOG responses in control and Skn-1a^-/-^ mice housed under our standard conditions. ***A***, Schematic drawing of a mouse heminose, showing the region where the EOG responses were recorded in the olfactory turbinate II. MOE, main olfactory epithelium; OB, olfactory bulb; VNO, vomeronasal organ; SO, septal organ. ***B***, ***C***, Representative images of MOE from control and Skn-1a^-/-^ mice, respectively, approximately corresponding to the region of EOG recording. The sections were stained with DAPI and imaged with additional weak transmitted light to review the morphology of the MOE in the area of olfactory turbinate II (OT II). Scale bar: 100 µm. ***D***, Representative EOG traces. ***E***, Average peak EOG responses to various stimuli (*n* = 6, mean ± SEM). Chemical stimuli were applied in exactly the same sequence for EOG recordings for both control and Skn-1a^-/-^ mice. The EOG responses to various odorants and pheromones at 100 µm were recorded first, followed by responses to the adenylyl cyclase activator forskolin (1 µm). Finally, the EOG responses to 2,5-dimethylpyrazine (2,5-DMP) and 2-heptanone (10 and 200 µm) were recorded. There was no significant difference in EOG amplitude in response to the same odorant between Skn-1a^-/-^ and control mice, except 2-heptanone at 200 µm (*, *p* < 0.05, *t* test, *n* = 6, mean ± SEM).

The method was adapted from a previous publication (Lin et al., 2008a). Briefly, mice were euthanized by CO_2_ inhalation followed by cervical dislocation. To minimize blood in the nasal tissue, exsanguination was performed through an open heart immediately after death. The skinned head was then split along the midline, and the nasal septum was removed to expose the olfactory turbinate. The half head was mounted on a recording chamber using low-melting-point agarose (2%). Ringer’s solution and odorants were delivered through a gravity-fed computer-controlled perfusion system with an approximate flow rate of 0.2 ml/s. Each odorant was presented three times (1-s duration, 1-min interval), and the largest response amplitude among the three repeats was measured for data analysis. The recording electrode was placed on the apical surface of endoturbinate II, and the reference Ag/AgCl electrode was placed in bath saline. The glass pipette recording electrode was made using a pipette puller (PP-830, Narishige), and the tip of the pipette was fire polished by a microforge (MF-83, Narishige) and filled with 0.9% agar made in Ringer’s saline with 1% neutral red. EOG recordings were made at the position of olfactory turbinate II (see Fig. 1*A*) using a differential amplifier (DP-311, Warner Instruments), a digital I/O and A/D controller (Instrutech ITC-18, Heka Elektronik), a solenoid valve for odor delivery, and a photo-coupler relay controlled by a personal computer with AxoGraph software. The recorded signals were digitized at 500 Hz and analyzed using AxoGraph software.

### Immunohistochemistry

#### Tissue preparation

We followed the immunolabeling procedure used for the initial characterization of TRPM5-MCs (Lin et al., 2008a and 2008b; Ogura et al., 2011). Briefly, mice from standard housing and 2-wk exposure groups were deeply anesthetized with tribromoethanol (Avertin; 250 µg/g body weight), perfusion-fixed with a phosphate-buffered fixative containing 3% paraformaldehyde, 19 mm l-lysine monohydrochloride, and 0.23% sodium m-periodate. The nose was harvested and postfixed for 1.5 h before being transferred to 0.1 m PBS with 25% sucrose overnight for cryoprotection. The nose was then manually deboned following a method described in Dunston et al. (2013), and MOE tissue was embedded. Tissue was then cut into 14-µm-thick sections using a cryostat (Microm International) and mounted onto Superfrost Plus microscope slides (Thermo Fisher Scientific). The slides were stored in a –80°C freezer until use.

#### Immunohistochemistry

MOE sections were rinsed in 0.1 m PBS three times, 10 min each, before incubating in PBS-buffered blocking solution containing 2% normal donkey serum, 0.3% Triton X-100, and 1% bovine serum albumin for 1.5 h. Sections were then immunoreacted for 48–72 h at 4°C with primary antibodies against olfactory marker protein (OMP, 1:1000, Wako 019-22291-WAKO RRID:AB_664696) and growth-associated protein 43 (GAP43, 1:2000, Novus NB300-143 RRID:AB_10001196). After incubation with the primary antibodies, sections were washed and reacted with secondary antibodies conjugated with either Alexa Fluor 555 or 647 (1:400; Invitrogen) for 1 h at room temperature. Sections were then rinsed and coverslipped with Fluoromount-G containing DAPI, which stains nuclei (Southern Biotech). In control experiments, primary antibodies were omitted, which resulted in negative labeling.

#### Image acquisition

Low-magnification images of the MOE were taken using an Olympus BX 41 epifluorescence compound microscope, equipped with a Retiga 4000R camera (QImaging), and acquired with Q-Capture Pro 7 (QImaging). High-magnification confocal images of immunolabeled sections were taken using an Olympus BX 61 epifluorescence microscope equipped with a spinning disk confocal unit and Slidebook 5.0 software (3i).

### Buried food test

This protocol was adapted from previous publications (Wersinger et al., 2007; Yang and Crawley, 2009). In this test, mice are required to dig to locate a piece of food that is buried under the bedding. To diminish avoidance of novel food during the test, mice were given a small piece of an Oreo cookie for two to three consecutive nights before the experiment. The night before testing, mice were food-deprived overnight for 14–19 h to increase their interest in food. A small piece of an Oreo cookie (∼70–110 mg) was buried under a layer of bedding 5–6 cm thick in a 16 × 15-cm search space within a standard mouse cage (see Fig. 4*A*). Mice were placed in the cage and allowed to freely explore the cage for 5 min (maximum cutoff). Mouse behavior was videorecorded from the side view to score the latency to locate the buried food. The mouse was considered to have successfully located the buried food when either its paws or snout reached within 1 cm of the cookie piece. Any animals that took longer than the cutoff time were excluded from further data analysis. One of a total of 13 mice from the group of control mice exposed to chemicals and two of a total of 14 mice from the water-exposed Skn-1a^-/-^ mice group were excluded for this reason. After the test, the mouse was returned to its home cage and supplied with food and water.

### T-maze odor choice test

A T-maze apparatus was used to assess the ability of male mice to detect socially and sexually relevant odors (Kavaliers et al., 1994). The transparent polycarbonate plastic T-maze apparatus consists of two arms (40 cm length and 10 cm width) with a short start arm (25 cm length; see Fig. 5*A*). The start arm has a movable gate to allow a mouse to access either the left or right arm, which avoids counting the time the mouse spends in the unclassified area between the two testing arms. The mice explore the T-maze based on olfactory cues presented only at the end of the arms. To eliminate visual cues, the experiment was performed in the dark, and mouse behavior was videorecorded under infrared lighting (Foscam F18918W). For the experiment, a single mouse was placed in the start arm while the gate was closed, which prevented it from entering the arms. After a 5-min acclimation, the gate was opened and the mouse was allowed to freely explore the maze for 10 min. Water and (female) urine samples (200 µl) were placed at the very end of the left and right arms randomly (1 cm from the wall) in a 35-mm Petri dish. At the end of the experiment, the mouse was returned to its home cage, and the whole apparatus was manually cleaned with 15% ethanol and dried with a fan to blow out any residual odor. The locations of the odor samples were counterbalanced between mice to eliminate possible location preference, and new samples were used for every trial. Mouse movements between the choice arms were evaluated in terms of approach behaviors when orienting toward the odor sources. When a mouse spent time in proximity to either Petri dish, it was considered to be sniffing the samples. Proximity was defined as the mouse’s snout coming within 1 cm of a Petri dish. JWatcher software (UCLA and Macquarie University) was used to quantify these parameters. The urine preference ratio was calculated by dividing the percentage of time spent in sniffing the urine dish by the total time spent sniffing both water and urine dishes.

### Block test to differentiate body odors

A modified version of the block test was conducted to assess olfactory-guided preference behavior based on the detection and discrimination of social odors (Tillerson et al., 2006; Fleming et al., 2008; Lehmkuhl et al., 2014). Naive male mice were individually housed in clean cages overnight (∼18 h) with four wood blocks [labeled A–D; (15 mm)^3^] placed inside the cage to obtain the individual mouse’s body odor. The following day, 1 h before beginning the experiment, these self-scented blocks were removed from the animals’ cages and kept in Ziploc bags with bedding for the duration of the experiment. The test consisted of three 2-min trials, separated by a ∼15- to 20-min intertrial interval. Individual mice naive to the assay were acclimated in their individual standard shoebox cages for 1 h before testing. The same cages were used for the test. At the beginning of each trial, the four blocks with the same scent cues (self body odor) were placed into the test cage with as little disturbance as possible (see Fig. 6*A* for setup). Blocks were placed in a random order with the letters facing the camera. In the first two trials, the self-scented blocks labeled A–D were presented. In the third trial, block D was replaced with a new block (E) with odor of a stranger male mouse. During trials, the mouse explored the blocks freely, and all tests were videorecorded for later analysis. An experimenter who was blind to genotype used JWatcher to determine the duration investigating the blocks and the number of approaches to each block. Approaches were defined as any movement that brought the mouse’s nose into close physical proximity (less than 1 cm) with a block. The mouse was considered to be sniffing when the mouse’s snout came into the close proximity with the blocks. Inactive mice that fixated on one block or failed to approach the blocks at least three times during the 2-min trial were excluded from data analysis.

### Statistical analysis

All data are presented as mean ± SEM. Student’s *t* test was used to compare results between the two experimental groups (chemical-exposed versus water-exposed). If the *F* test was significant and homogeneity of variance was not assumed, Welch’s *t* test was used instead of Student’s *t* test. For the approach data from the block test, a comparison of the number of approaches for each block was made by a one-way ANOVA. Bonferroni corrections were performed for the *post hoc* comparisons as needed. For all tests, *p* < 0.05 was considered to be statistically different. During our study, a report about the relatively higher metabolism of Skn-1a^-/-^ mice was published (Ushiama et al., 2016). For this reason, we compared data only from animals that were paired, i.e., vehicle- versus chemical-exposed animals within the same mouse line.

## Results

### Skn-1a^-/-^ mice do not show altered olfactory responses when housed under standard conditions

We first examined whether odor-evoked EOG responses are altered in Skn-1a^-/-^ mice lacking TRPM5-MCs in the MOE, since physiological characterization of the impact of Skn-1a knockout in the olfactory system has not previously been performed. For olfactory stimuli, we used citral, geraniol, acetophenone, and amyl acetate as common volatile odorants. We also included propionic acid and triethylamine, which were present in our odor mixture for the 2-wk exposure. Propionic acid was selected because it can be produced by bacteria commonly found in nasal mucosa (Boase et al., 2013), and TRPM5-MCs respond to bacterial lysate (Ogura et al., 2011). Triethylamine was chosen for its widespread use in chemical synthesis. Additionally, synthetic urinary pheromones 2-heptanone and 2,5-dimethylpyrazine (2,5-DMP) and the adenylyl cyclase activator forskolin were included as MOE stimuli. EOGs were recorded from the olfactory turbinate II as indicated (Fig. 1*A*). Histologic examination of MOE sections obtained from the same region showed similar morphology between control TRPM5-GFP and Skn-1a^-/-^ mice (Fig. 1*B* and *C*, respectively). We found that control and Skn-1a^-/-^ mice housed under standard conditions responded to all volatile stimuli (100 μM) and forskolin (1 μM) tested during EOG recordings (Fig. 1*D*: representative EOG traces from control and Skn-1a^-/-^ mice). Responses obtained from Skn-1a^-/-^ mice trended slightly higher for most of the stimuli but were not significantly different from the EOG responses of control mice (Fig. 1*E*; *t*(9) = 0.217–1.387, *p* = 0.1–0.3, *n* = 5 and 6, control and Skn-1a^-/-^ mice), except for 2-heptanone (200 μM; *t*(7) = 3.669, *p* = 0.004; Fig. 1*E*: plot of average response amplitude to individual stimuli). Forskolin did not cause any difference in EOG between control and Skn-1a^-/-^ mice. We also observed no apparent differences in the response kinetics, such as the rates of activation and recovery between control and Skn-1a^-/-^ mice (data not shown). Therefore, the lack of TRPM5-MCs in the MOE of Skn-1a^-/-^ mice does not have a significant impact on evoked olfactory responses to select odorants and pheromones under standard housing conditions.

### Reduced odor-evoked EOG responses in Skn-1a^-/-^ mice after a 2-wk chemical exposure

**Figure 2.**
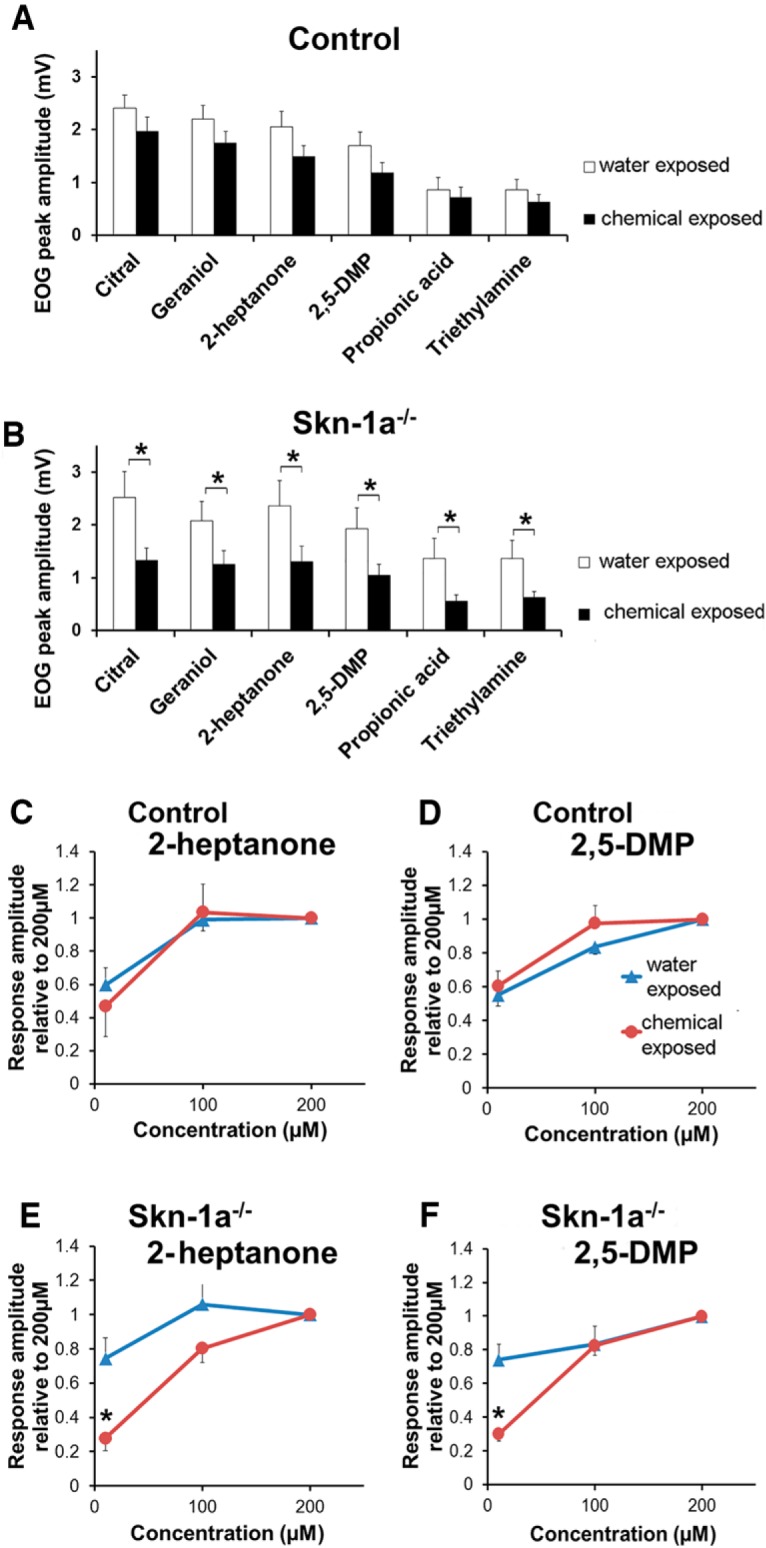
EOG responses in chemical-exposed Skn-1a^-/-^ mice are significantly reduced. ***A***, Average peak EOG responses to odorants and pheromones at 100 µm in control mice. There was no significant difference in the response amplitude to the same stimuli between water- and chemical-exposed groups (*p* > 0.05, *t* test, *n* = 15 and 16, respectively). ***B***, Average peak EOG responses to odorants at 100 µm in Skn-1a^-/-^ mice. The EOG responses obtained from the chemical-exposed group were significantly smaller than those from the water-exposed group (*, *p* < 0.05 *t test*, *n* = 15 and 18, respectively). ***C–F***, Normalized EOG responses to 2-heptanone or 2,5-DMP at various concentrations (10, 100, and 200 µm), which are presented relative to the values of EOG responses at 200 µm of the same animals. There was no significant difference in the dose-dependent responses between the water- and chemical-exposed groups in control mice (*p* > 0.05, *n* = 4–6). In Skn-1a^-/-^ mice, normalized responses at 10 µm in chemical-exposed group are significantly smaller than responses of the vehicle group (*, *p* < 0.05, *t* test, *n* = 5–6).

Previously, TRPM5-MCs were found to be responsive to odorous stimuli at relatively high concentrations and capable of releasing acetylcholine (ACh), which modulates intracellular Ca^2+^ levels in OSNs and supporting cells (Ogura et al., 2011). These findings have led to a hypothesis that TRPM5-MCs are involved in the maintenance of MOE function in challenging chemical environments. To test this, we performed EOG recordings in control and Skn-1a^-/-^ mice after they had been exposed to a mixture of chemicals and chitin for 2 wks. We found that EOG responses in chemical-exposed control TRPM5-GFP mice trended lower compared with the water-exposed mice. However, the reduction was not significant for odorants citral and geraniol and pheromones 2-heptanone and 2,5-DMP (Fig. 2*A*, *t*(29) = 1.209–1.602, *p* = 0.1–0.2, *n* = 15 and 16, water and chemical-exposed, respectively), as well as for the two chemical exposure compounds propionic acid and triethylamine (Fig. 2*A*; *t*(29) = 0.451–0.917, *p* = 0.1–0.2, *n* = 15 and 16, water and chemical-exposed, respectively). In contrast, in chemical-exposed Skn-1a^-/-^ mice, the average EOG amplitudes were significantly reduced for most of the stimuli, including odorants, pheromones, and chemicals in the exposure mixture, compared with the Skn-1a^-/-^ mice of the water-exposed group (Fig. 2*B*; *t*(31) = 1.852–2.351, *p* = 0.019–0.041, *n* = 15 and 18, water and chemical-exposed, respectively). Therefore, these EOG results obtained after chemical exposure indicate an important role of TRPM5-MCs in maintaining the olfactory responses of the MOE.

We also investigated the impact of chemical exposure on dose-dependent responses to 2-heptanone and 2,5-DMP (10, 100, and 200 µm), since these volatile urinary pheromones were most likely present in their housing environment. Reduced EOG responses were found at all three concentrations in the chemical-exposed groups, with a larger reduction in Skn-1a^-/-^ mice, as expected. When the responses were normalized to the peak response values obtained at 200 µm from the same animals, we found that the dose–response curves for these two pheromones were similar between the chemical- and vehicle-exposed control mice (Fig. 2*C*, *D*; *t*(df) = 0.173–1.423, *p* = 0.173–1.425 for both 10 and 100 µm, *n* = 4–6). In striking contrast, Skn-1a^-/-^ mice in the chemical exposure group showed significantly smaller responses to these stimuli at 10 µm concentration compared with those obtained from the water-exposed Skn-1a^-/-^ mice (Fig. 2*E*, *F*; *t*(df) = 3.180 and 3.315 for 2-heptanone and 2,5-DMP, respectively, *p* = 0.006 and 0.005, *n* = 5–6), indicating impaired sensitivity to urinary pheromones in Skn-1a^-/-^ mice after chemical exposure. Together, these results suggest that TRPM5-MCs are important for the MOE to maintain its functional integrity.

### Two-week chemical exposure does not significantly alter the morphology of the posterior MOE

**Figure 3.**
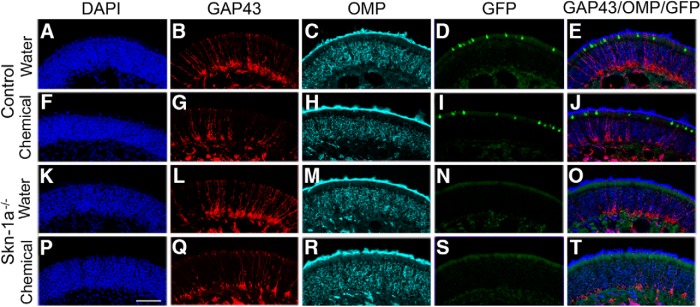
Immunohistochemical examination of the posterior MOE in control and Skn-1a^-/-^ mice after 2-wk exposure. Posterior MOE sections at approximately the same region where EOG was recorded were obtained from control and Skn-1a^-/-^ mice exposed to either water or chemicals for 2 wks. The sections were immunoreacted with antibodies against GAP43 and OMP and stained with DAPI. Confocal images were taken from olfactory turbinate II. ***A–E***, Control, water-exposed. ***F–J***, Control, chemical-exposed. ***K–O***, Skn-1a^-/-^ mouse, water-exposed. ***P–T***, Skn-1a^-/-^ mouse, chemical-exposed. DAPI staining (blue; ***A***, ***F***, ***K***, ***P***); GAP43 immunoreactivity (red; ***B***, ***G***, ***L***, ***Q***); OMP immunoreactivity (cyan; ***C***, ***H***, ***M***, ***R***); GFP-positive TRPM5-MCs (green; ***D*** and ***I*** only, control mice); overlay of GAP 43, OMP, and GFP (***E***, ***J***, ***O***, ***T***). Similar morphology and marker expression were found in four groups of mice, indicating there was no obvious tissue damage in both control and Skn-1a^-/-^ mice after chemical exposure in the posterior MOE regions where EOG recordings were performed. Scale bar: 50 µm.

The deficit in evoked odor responses in chemical-exposed Skn-1a^-/-^ mice could result from impaired functional modification or damage to the MOE tissue. The MOE thickness varies substantially depending on regions under normal conditions. We performed immunohistochemical examination of MOE sections obtained from the approximate region where the EOG responses were recorded using antibodies against GAP43 and OMP, which label immature and mature OSNs, respectively. The sections were also stained with DAPI to view the nuclei. We found that the MOE of turbinate II from mice of all four groups exhibited similar morphology, with a smooth surface and no apparent swelling of DAPI-labeled nuclei, indicating that the 2-wk chemical exposure caused minimal or no tissue damage in this region for either control TRPM5-GFP or Skn-1a^-/-^ mice (Fig. 3*A*, *F*, *K*, *P*; confocal images of DAPI staining). Similar labeling for GAP43 and OMP was also seen in these regions (Fig. 3*B*, *G*, *L*, *Q* for GAP43; Fig. 3*C*, *H*, *M*, *R* for OMP). Furthermore, there was no drastic change in the number of TRPM5-MCs (GFP positive, Fig. 3*D*, *I* from water- and chemical-exposed, control TRPM5-GFP mice, respectively; Fig. 3*E*, *J*, *O*, *T*: overlay of GAP 43, OMP, and GFP). Therefore, impairment in functional modification rather than tissue damage most likely underlies the significant reduction in the EOG responses observed in chemical-exposed Skn-1a^-/-^ mice.

### Skn-1a^-/-^ mice exhibit compromised olfactory ability to locate buried food after a 2-wk chemical exposure

**Figure. 4.**
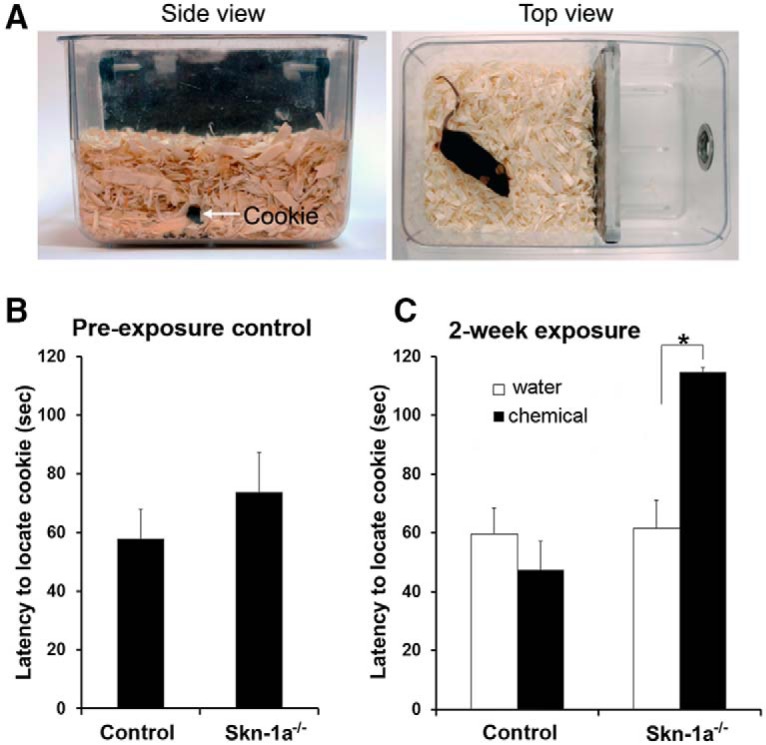
Impaired olfactory ability to locate buried food in Skn-1a^-/-^ mice after chemical exposure. ***A***, Photographs of the experimental setting for the buried food test. Left panel: side view, showing 5- to 6-cm wood chip bedding over a buried cookie piece (indicated by an arrow). Right panel: top view before a mouse began to search for the piece of cookie. ***B***, ***C***, Plot of the average time (latency) required for mice to locate the buried cookie. The latency was not significantly different between control and Skn-1a^-/-^ mice housed under our standard conditions (***B****; n* = 14 and 13, WT and Skn-1a^-/-^, respectively). After chemical exposure, Skn-1a^-/-^ mice took a significantly longer time to locate the buried cookie compared with the water-exposed group (*p < 0.05, *t test*, *n* = 14 and 9, respectively). There was no difference between the water- and chemical-exposed groups of control mice (***C***; *n* = 9 and 12, respectively).

We next investigated whether the reduction in odor-evoked EOG responses in the chemical-exposed Skn-1a^-/-^ mice leads to deficits in olfactory-guided behaviors. Fig. 4*A* shows the setup for assessing the animals’ general olfactory ability to locate buried food. To avoid learning-related complications, we performed this assay using only naive mice that had never been previously tested for finding buried cookies. We first assessed whether Skn-1a^-/-^ and control mice, which were housed under our standard conditions without the chemical exposure, differed in the time required to locate the buried cookie. We found there was no difference in their performance (Fig. 4*B*; *t*(df) = 25, *p* = 0.175, *n* = 13 and 14, respectively).

We performed the same test on control and Skn-1a^-/-^ mice after 2-wk water or chemical exposure. Chemical-exposed control mice showed no difference in performance compared with water-exposed mice (Fig. 4*C*; *t*(19) = 0.903, *p* = 0.189, *n* = 9 and 12, water- and chemical-exposed, respectively), indicating that chemical exposure did not have an adverse impact on olfactory-guided food searching behavior in control mice. In contrast, chemical-exposed Skn-1a^-/-^ mice took significantly longer to locate a buried cookie than water-exposed Skn-1a^-/-^ controls (Fig. 4*C*, *t*(19.9) = 2.424, *p* = 0.013, *n* = 9 and 14, water- and chemical-exposed, respectively). These data indicate that the olfactory ability of guiding food-finding was impaired in Skn-1a^-/-^ mice, but not in control mice after chemical exposure.

### Olfactory preference toward urine of the opposite sex is reduced in Skn-1a^-/-^ mice after 2-wk chemical exposure

**Figure 5.**
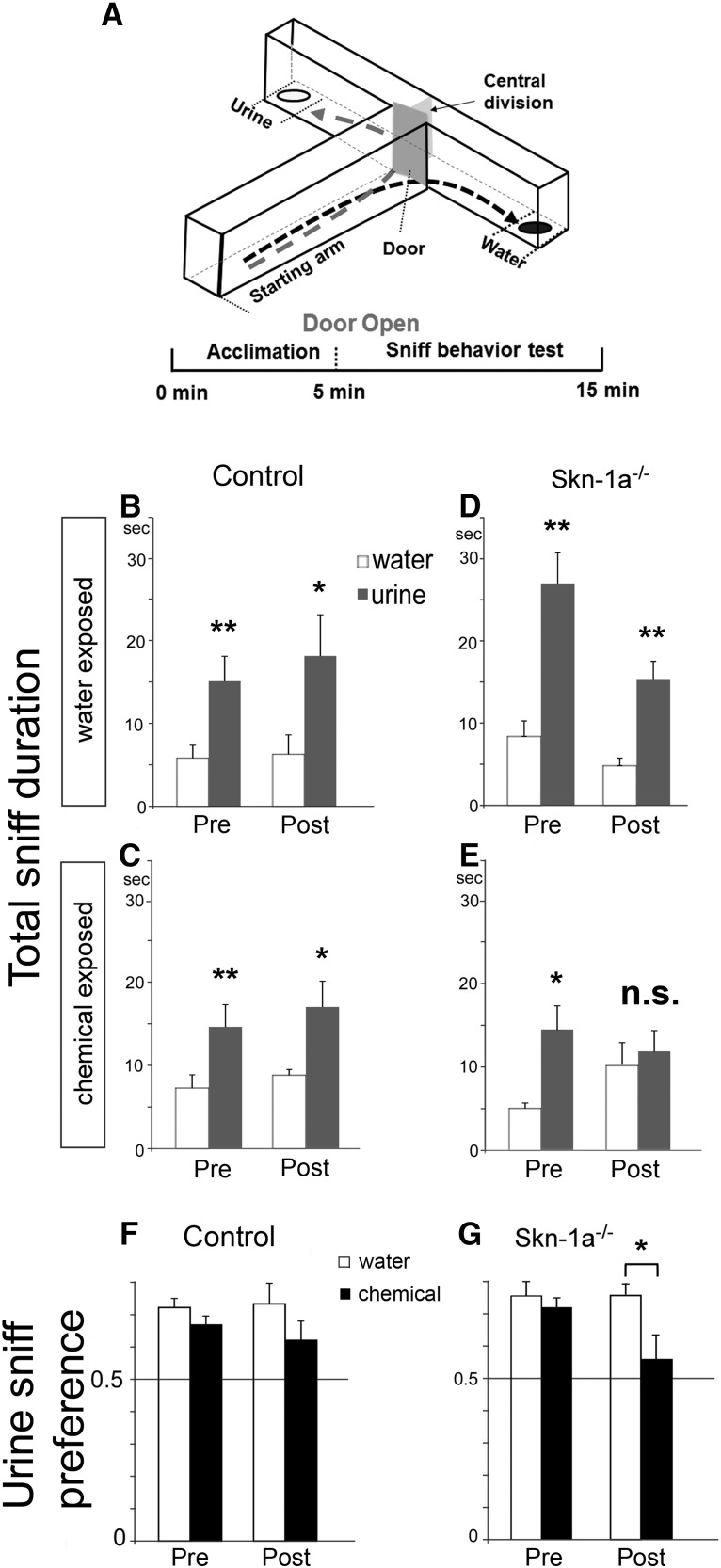
Olfactory preference toward urine of the opposite sex is compromised in chemical-exposed Skn-1a^-/-^ mice. ***A***, Schematic drawing of the T-maze apparatus used to test olfactory preference for urine. A single mouse was placed in the starting arm of the T-maze for a 5-min acclimation period before the gate was opened, which allowed the mouse to enter either arm. ***B–E***, Plots of total sniff duration. Both control and Skn-1a^-/-^ male mice displayed a strong preference for the female urine samples before exposure (*, *p* < 0.05, **, *p* < 0.01, paired *t* test, *n* = 5–8). Control mice retained their strong preference for urine samples after exposure to either water or chemicals (*, *p* < 0.05, **, *p* < 0.01, paired *t* test, *n* = 5–8). In contrast, only the water-exposed Skn-1a^-/-^ mice maintained the strong preference (**, *p* < 0.01, paired *t* test, *n* = 6). Chemical-exposed Skn-1a^-/-^ mice no longer significantly preferred urine over water (*p* = 0.316, paired *t* test, *n* = 7). ***F***, ***G***, Plots of sniff preference (ratio of urine sniff duration over total sniff duration). No significant difference was found in the control mice pre- and post-exposure (*p* = 0.113 and 0.120, respectively, *t* test), or the pre-exposure Skn-1a^-/-^ mice group (*p* = 0.207, *t* test). However, a significant reduction was found in Skn-1a^-/-^ mice of chemical-exposed group compared with the water-exposed group (*, *p* < 0.05, *t* test).

In addition to sensing common airborne odorants, the MOE and its associated main olfactory system play an important role in detecting and discriminating semiochemicals and providing guidance for social and sexual behaviors (Keverne, 2004; Mandiyan et al., 2005; Lin et al., 2007; Fraser and Shah, 2014; Lopez et al., 2014). Semiochemicals or social/sexual odors present in body secretions, such as urine, contain rich information about the genetic background as well as social and sexual status of the animals (Boehm and Zufall, 2006; Brennan and Zufall, 2006; Restrepo et al., 2006; Peretto and Paredes, 2014). Generally, male mice are keen to investigate female urine over water (Achiraman and Archunan, 2002). We investigated whether chemical exposure would compromise the detection of urine from the opposite sex (female) using a T-maze choice test (Fig. 5*A*). Individual male mice in these groups were tested before and after the 2-wk exposure. Both control and Skn-1a^-/-^ mice were clearly capable of discriminating between urine and water in the pre-exposure trial and preferred female urine, as shown by the higher duration of time spent sniffing the urine sample than water (Fig. 5*B*, control mice, pre-water exposure; *t*(4) = 4.386, *p* = 0.006, *n* = 5; Fig. 5*C*, control mice, pre-chemical exposure *t*(7) = 3.614, *p* = 0.004, *n* = 8; Fig. 5*D*, Skn-1a^-/-^ mice, pre-water exposure, *t*(5) = 4.115, *p* = 0.005, *n* = 6; Fig. 5*E*, Skn-1a^-/-^ mice, pre-chemical exposure *t*(6) = 2.574, *p* = 0.021, *n* = 7). After chemical exposure, control mice maintained the preference for urine over water (Fig. 5*B*, post-water exposure; *t*(5) = 3.307 *p* = 0.015, *n* = 5; Fig. 5*C*, post-chemical exposure *t*(7) = 2.714, *p* = 0.015, *n* = 8). However, olfactory detection and discrimination ability were impaired in chemical-exposed Skn-1a^-/-^ mice, as these mice no longer significantly preferred urine over water compared with the water-exposed Skn-1a^-/-^ mice (Fig. 5*D*, post-water exposure, *t*(5) = 5.646, *p* = 0.001, *n* = 6; Fig. 5*E*, post-chemical exposure *t*(6) = 0.504, *p* = 0.316, *n* = 7).

A further analysis of urine preference ratio confirmed the effect of chemical exposure. In the pre-exposure test, there were no significant differences in urine preference ratio between intended water- and chemical-exposed groups of either control mice (Fig. 5*F*, *t*(11) = 1.281 *p* = 0.113, *n* = 5 and 8) or Skn-1a^-/-^ mice (Fig. 5*G*, *t*(11) = 0.850, *p* = 0.207, *n* = 6 and 7). After exposure, chemical-exposed control mice did not show a significant reduction in the urine preference ratio compared with the water-exposed control mice (Fig. 5*F*, *t*(11) = 1.242, *p* = 0.120, *n* = 8 and 5). However, chemical-exposed Skn-1a^-/-^ mice showed a significant reduction in the urine preference ratio compared with the water-exposed Skn-1a^-/-^ mice (Fig. 5*G*, *t*(11) = 2.136, *p* = 0.028, *n* = 7 and 6). These results demonstrated that unlike control mice, Skn-1a^-/-^ mice were not able to maintain their olfactory ability of detecting odors from the opposite sex to guide their behavioral reaction after chemical exposure.

### Impaired detection of novel social odors in Skn-1a^-/-^ Mice after a 2-wk chemical exposure

**Figure 6.**
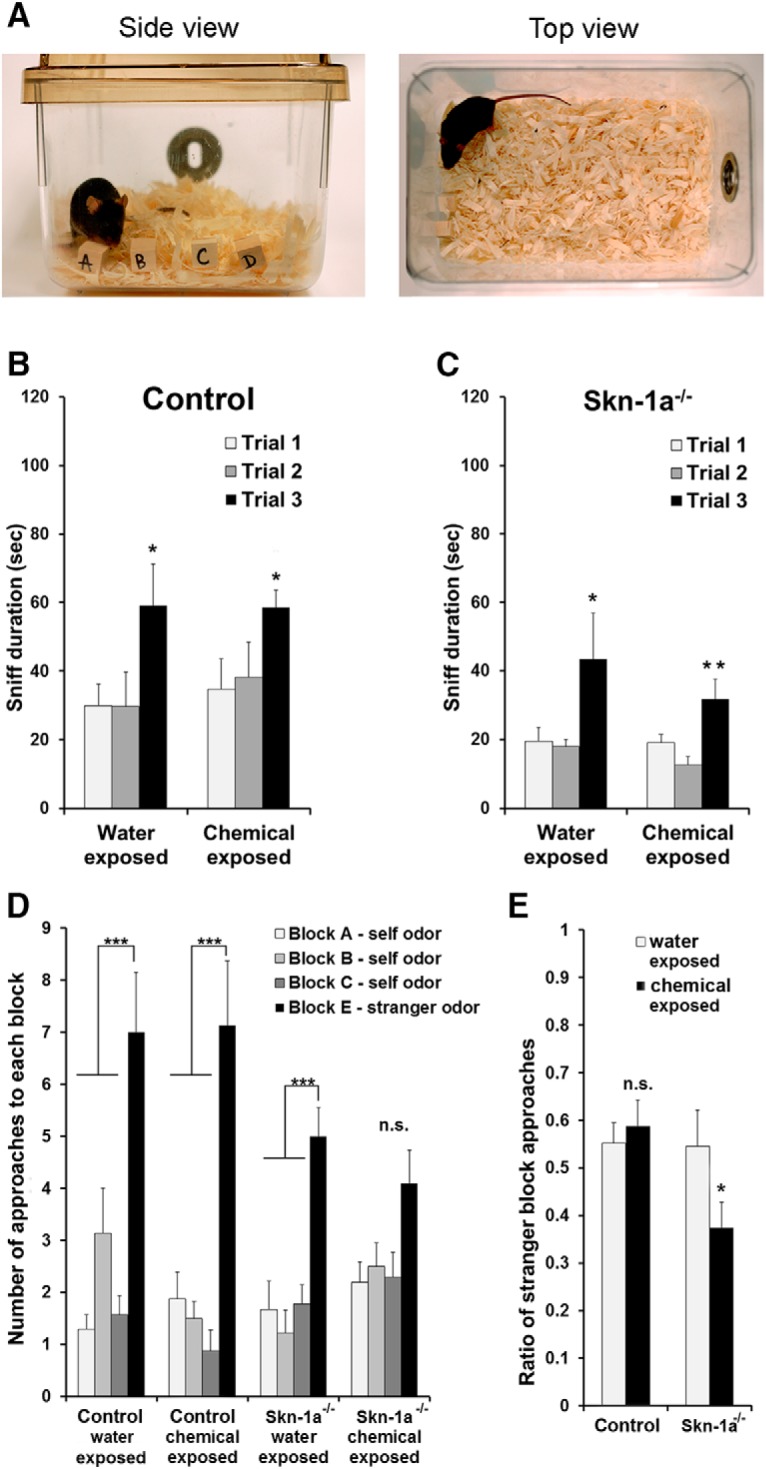
The number of approaches to a novel social odor is reduced in Skn-1a^-/-^ mice after chemical exposure. ***A***, Photographs of the experimental setting for the block test. Four scented blocks were placed into the cage. Left panel: side view, a mouse was sniffing block A. Right panel: top view. ***B***, Sniff duration for each trial in water- and chemical-exposed groups of control mice. There were three trials total with each lasting 120 s. All blocks in trials 1 and 2 were scented with the mouse’s own odors. In trial 3, the block D was replaced with a block scented with a stranger odor (block E). Control mice of both water- and chemical-exposed groups sniffed significantly longer in trial 3 (*, *p* < 0.05, trial 3 vs. trial 2, *t* test, *n* = 7–8). ***C***, Sniff duration for each trial in water- and chemical-exposed groups of Skn-1a^-/-^ mice. Skn-1a^-/-^ mice in both conditions displayed significantly longer sniff duration in trial 3 (*, *p* < 0.05, **, *p* < 0.01, trial 3 vs. trial 2, *t* test, *n* = 9–10). ***D***, Number of approaches to each block during trial 3 in water- or chemical-exposed groups of control and Skn-1a^-/-^ mice. In control mice, both water- and chemical-exposed groups approached the block scented with a stranger odor significantly more than the three blocks scented with the mouse’s own odors (***, *p* < 0.0001, one-way ANOVA, *n* = 7 and 8). Similarly, water-exposed Skn-1a^-/-^ mice also approached the stranger odor block significantly more (***, *p* < 0.0001, one-way ANOVA, *n* = 9). However, chemical-exposed Skn-1a^-/-^ mice failed to show significantly more approaches to the stranger scented block compared with other blocks scented with their own odors, although the number trends higher [one-way ANOVA (*p* = 0.0341), Bonferroni corrections vs. the stranger scent block (*P*s > 0.05)]. ***E***, Comparison of preference index. The index was calculated using the number of approaches to the stranger scented block versus the number of approaches to all blocks. Although the preference index is very similar between the water- and chemical-exposed groups of control mice, the ratio of chemical-exposed Skn-1a^-/-^ mice was significantly lower than the ratio of water-exposed Skn-1a^-/-^ mice (*, *p* < 0.05, *t* test, *n* = 9 and 10).

Finally, we examined the olfactory ability of Skn-1a^-/-^ and control mice to detect and discriminate social odors by assessing their behavior toward the body odor of a stranger mouse versus their own body odor, using a modified version of the block test described previously (Fleming et al., 2008; Lehmkuhl et al., 2014; Tillerson et al., 2006). During the first two trials of this assay, when individual mice were presented with four wooden blocks scented with their own body odors (Fig. 6*A*), both control and Skn-1a^-/-^ mice exposed to either water or chemicals were interested in blocks scented by their own odors and sniffed the individual blocks readily (Fig. 6*B*, *C*: total sniff durations for trial 1 and 2 of control and Skn-1a^-/-^ mice, respectively). In the third trial, when a block with the mouse’s own scent was replaced with a block scented with a stranger’s odor, both control and Skn-1a^-/-^ mice exposed to either water or chemicals showed a strong interest in the block with the stranger’s odor, as evidenced by significant increases in the total sniffing duration (including stranger scent) in trial 3 compared with trial 2 (own scent only; control, water, *t*(13) = 1.889, *p* = 0.041, *n* = 7 and 8; control, chemical, *t*(13) = 1.799, *p* = 0.048, *n* = 7 and 8; Skn-1a^-/-^, water, *t*(16) = 1.855, *p* = 0.041, *n* = 9; Skn-1a^-/-^, chemical, *t*(17) = 2.895, *p* = 0.005, *n* = 9 and 10). The increased duration of sniffing the blocks indicates that these mice were capable of detecting and discriminating social odors and were motivated to investigate semiochemicals embedded in the scented blocks.

Both the MOE and vomeronasal organ (VNO) sense social odors. However, the MOE is often required for detecting the volatile components in social odor blends and guiding the animal to the stimulus source (Keller et al., 2006; Slotnick et al., 2010). Given the sequential process of olfactory behavior, we closely examined the number of approaching/sniffing events. During a typical sniffing event, mice often stretch their bodies toward the stimulus to approach and assess it and then quickly retreat/draw back (Blanchard et al., 2011). We counted the number of assessment events (approaching) to evaluate how control and Skn-1a^-/-^ mice differentiated the stranger-scented block from self-scented blocks. Fig. 6*D* depicts the mean number of approaches to each block in control and Skn-1a^-/-^ mice that were exposed to water or chemicals in trial 3. One-way ANOVA on the number of approaches to the blocks (A, B, and C blocks, own odor; E block, stranger odor) revealed an asymmetry profile of the proportion of block approaches in both control and Skn-1a^-/-^ mice. Control mice exposed to either water or chemicals exhibited a greater number of approaches to the stranger-scented block compared with self-scented blocks (control, water-exposed, *F*(3,24) = 12.10, *p* < 0.0001, *n* = 7; control, chemical-exposed *F*(3,28) = 15.97, *p* < 0.0001, *n* = 8). Similarly, Skn-1a^-/-^ mice exposed to water displayed more approaches to the stranger scent block compared with other self-scented blocks (water-exposed, *F*(3,32) = 12.98, *p* < 0.0001, *n* = 9), whereas Skn-1a^-/-^ mice that were exposed to chemicals failed to show a significant increase in the number of approaches to the stranger-scented block compared with self-scented blocks (chemical-exposed, *F*(3,36) = 3.215, *p*= 0.0341, *n* = 10, Bonferroni corrections vs. the stranger-scented block *P*s > 0.05). These results indicate that control mice were able to maintain their olfactory ability of discriminating social odors (self vs. stranger), whereas this ability was impaired in Skn-1a^-/-^ mice after chemical exposure.

We calculated the ratio of the number of approaches to the stranger-scented block over the total number of times approaching all blocks in control and Skn-1a^-/-^ mice exposed to water or chemicals (Fig. 6*E*). Control mice displayed a similarly high ratio of approaches to the stranger block regardless of exposure condition (*t*(13) = 0.502, *p* = 0.312, *n* = 7 and 8). In contrast, Skn-1a^-/-^ mice that were exposed to chemicals showed a significant decrease in approach ratio compared with those that were exposed to water (*t*(17) = 1.870, *p* = 0.039, *n* = 10 and 9). Altogether, our analysis of sniffing behavior in the block test indicates the significant impairment of social odor discrimination in Skn-1a^-/-^ mice after chemical exposure.

## Discussion

The use of Skn-1a^-/-^ mice in our physiological recordings and behavioral assays has enabled us to elucidate the function of TRPM5-MCs in the MOE. We found that the EOG responses evoked by odorants and pheromones in Skn-1a^-/-^ mice were significantly reduced after 2-wk exposure to a mixture of volatile chemicals at relatively high concentrations and chitin powder compared with water-exposed Skn-1a^-/-^ mice. We also found that chemical-exposed Skn-1a^-/-^ mice were behaviorally compromised in their olfactory-guided food-finding and reactions toward socially and sexually relevant odors. These significant physiologic and behavioral deficits found in Skn-1a^-/-^ mice were not observed in control mice treated under the same conditions. Considering that Skn-1a^-/-^ mice lack TRPM5-MCs, these results strongly suggest that TRPM5-MCs play an important role in maintaining the olfactory function of the MOE and subsequently ensuring the maintenance of olfactory-guided behaviors under a challenging chemical environment. Therefore, our results reveal a novel mechanism for protective regulation of MOE activity during chemical insult.

The TRPM5-MC–mediated protective regulation we observed in this study can be distinguished from sensory adaption within the OSNs. In sensory adaptation, physiologic responses of OSNs to prolonged odor stimulation are decreased over time via intracellular Ca^2+^-mediated down-regulation of olfactory signal transduction, active Ca^2+^ clearance, and decreased expression of cognate odorant receptor genes to the stimulus (Munger et al., 2001; Stephan et al., 2012; von der Weid et al., 2015; Ferguson and Zhao, 2017). Thus, sensory adaptation occurs in specific OSNs that express odorant receptors to the exposed stimulus. However, chemical-exposed Skn-1a^-/-^ mice responded significantly less not only to the chemicals used for chronic exposure, but also to structurally unrelated chemicals and pheromones, suggesting that olfactory sensation is generally compromised. Therefore, it is unlikely that sensory adaptation is the primarily mechanism responsible for the phenotypes observed in our experimental conditions.

What mechanisms are likely to underlie the general compromise in odor responses of Skn-1a^-/-^ mice during chronic chemical exposure? It is unlikely that tissue damage was a major contributing factor, because similar morphology and cell marker expression were found in the MOE lining the olfactory turbinate of water- and chemical-exposed Skn-1a^-/-^ mouse groups. Skn-1a is expressed in the TRPM5-MCs and a minor subset of olfactory progenitor neurons (Yamaguchi et al., 2014). Even if Skn-1a were necessary for the functional differentiation of some OSNs, their population should be very limited. The loss of those OSNs or their function due to Skn-1a deficiency should not cause such a general olfactory deficit during chemical exposure. This explanation is supported by our data obtained from Skn-1a^-/-^ mice housed under our standard conditions, which did not show deficits in EOG responses to the set of odorants and pheromones tested. Rather, the loss of TRPM5-MCs can account for the compromised odor responses of Skn-1a^-/-^ mice during chemical exposure. As reported by Ogura et al. (2011), TRPM5-MCs can be activated by a variety of chemicals including odorants at high concentrations, ATP, and bacterial substances. TRPM5-MCs are also capable of synthesis and release of ACh, which potently increases intracellular Ca^2+^ levels in neighboring SCs and suppresses Ca^2+^ increases induced by the adenylyl cyclase activator forskolin in some OSNs due to differential expression of muscarinic ACh receptor (AChR) subtypes. Cholinergic enhancement of olfactory responses via M3 AChR-mediated modulation of odor receptor activation was recently reported by another group (Jiang et al., 2015). Therefore, cholinergic pathways in the MOE play an important role in modulating olfactory activity under various chemical environments.

Cholinergic paracrine regulation mediated by nonneuronal cell types within the MOE has not been investigated until now. TRPM5-MCs are responsive to odorous stimuli at the level of 500 µm and higher, with little sensitivity to odorants at low concentrations that are sufficient to stimulate OSNs (Ogura et al., 2011). Based on this fact and the phenotypes we observed in chemical-exposed Skn-1a^-/-^ mice, we speculate that cholinergic TRPM5-MC–mediated regulation is more tuned to functional maintenance when the MOE is challenged by high levels of chemical stimuli. This explanation is also supported by our results showing comparable EOG and behavior between control and Skn-1a^-/-^ mice under standard housing conditions. OSNs reportedly express M2 and M4 AChRs (Ogura et al., 2011). Activation of these inhibitory receptors leads to a decrease in the cAMP production via Gαi/o pathway (Felder, 1995), which in OSNs would reduce potential damage from Ca^2+^ overload caused by nonspecific stimulation by high levels of odorants. This intercellular mechanism is expected to affect the MOE activity broadly during chemical exposure and would not be limited to the specific OSNs that are especially sensitive to the exposure odorants.

The results obtained from our behavioral studies are in strong agreement with our physiologic findings. All three assays showed that the olfactory-guided behaviors of food searching and preferential reactions toward social and sexual odors are impaired in chemical-exposed Skn-1a^-/-^ mice. These significant behavioral impairments were not observed in chemical-exposed control mice. Because we also observed a significant reduction in evoked EOG responses to odorants and pheromones in chemical-exposed Skn-1a^-/-^ mice, we consider that these behavioral impairments result directly from their impaired MOE function. Recently, TRPM5 was reported to express in a small population of vomeronasal sensory neurons (VSNs; Kusumakshi et al., 2015). The function of these VSNs and whether they require Skn-1a for functional differentiation have not been determined. In our block test, chemical-exposed Skn-1a^-/-^ mice still spent significantly more time sniffing the novel block than the familiar blocks scented by their own odors. The sniffing duration may reflect chemical sensing by both the main and accessory olfactory systems, because both systems process semiochemical information in parallel, also implying that the VNO function is intact in Skn-1a^-/-^ mice. However, sensory detection via the VNO requires animals to contact the stimulus source closely so that nonvolatile semiochemicals can be taken into the VNO (Halpern and Martinez-Marcos, 2003; Luo et al., 2003). Because VSNs are not the primary neurons guiding the cognitive behavior that relies on olfactory cues (and active approaching requires chemical detection in the MOE; Keller et al., 2006; Slotnick et al., 2010), the significant reduction in the number of approaches to the stranger-scented block is evidence of impaired olfactory function of the MOE. In Skn-1a^-/-^ mice, TRPM5-expressing taste receptor cells in the tongue and solitary chemosensory cells in the gut and respiratory passageway are also eliminated (Matsumoto et al., 2011; Ohmoto et al., 2013; Gerbe et al., 2016). It remains to be determined whether the lack of TRPM5-expressing cells in other tissues would adversely impact olfactory-guided behaviors.

In both the natural environment and occupational settings, humans and animals are exposed to a wide range of chemicals either sporadically or chronically in countless combinations and conditions. Chronic and severe chemical exposure is known to induce rhinitis, olfactory dysfunction, and other respiratory illnesses (Doty and Mishra, 2001; Kerr, 2015), which may be because such conditions likely overpower the ability of TRPM5-MCs to maintain MOE function. Therefore, our intention for this study was to choose chemical exposure conditions, to the best of our knowledge, that would challenge but not overwhelm the MOE’s ability to maintain its integrity so that we could evaluate the role of TRPM5-MCs in epithelial maintenance. Our chosen chemical exposure conditions produced no obvious tissue damage in the MOE of olfactory turbinates (Fig. 3) and had only a minor effect on the olfactory function of control mice (Fig. 2). Because of the limited knowledge about these cells and the MOE chemical defense mechanisms, our conditions for chemical exposure might not be the optimal way to engage TRPM5-MCs. Also, in our physiologic recordings, we used individual odorants and pheromones to evoke EOG responses, whereas in our behavioral assays, complex olfactory cues from food odor, urine samples, and body scents guided the mouse behavior. Because we used mice of both sexes in our physiologic study, we chose to stimulate the MOE with synthetic urinary pheromones instead of diluted urine samples, which include hundreds of volatiles, semiochemicals, and ions at various concentrations (Schaefer et al., 2002). This strategy allowed us to avoid complications caused by sex-specific variations in urinary components and responses, thus reducing the number of mice used for this study. Future studies may need to evaluate the physiologic impact of chemical exposure and Skn-1a knockout on olfactory responses to complex odor blends. Despite the limitations, our results have uncovered a novel intercellular mechanism for maintaining MOE function. Additionally, our results provide new knowledge about the diverse and important roles of TRPM5-expressing chemosensory cells found in the respiratory, gastrointestinal, urinary tracts, etc. (Lin et al., 2008a; Ogura et al., 2010; Tizzano et al., 2010; Finger and Kinnamon, 2011; Krasteva et al., 2011; Deckmann et al., 2014; Gerbe et al., 2016; Howitt et al., 2016). Our current findings about the function of TRPM5-MCs are consistent with the overall physiologic functions of these TRPM5-expressing chemosensory cells.

In conclusion, we have identified novel TRPM5-MC–mediated protective regulation that enables the MOE to sustain its function in a relatively strong chemical environment without compromising olfactory-guided behaviors.

## Synthesis

Reviewing Editor: Douglas Bayliss, University of Virginia School of Medicine Decisions are customarily a result of the Reviewing Editor and the peer reviewers coming together and discussing their recommendations until a consensus is reached. When revisions are invited, a fact-based synthesis statement explaining their decision and outlining what is needed to prepare a revision will be listed below. The following reviewer(s) agreed to reveal their identity: Haiqing Zhao, Minghong Ma.

The two Reviewers of this manuscript concur that the study reveals a significant role of TRPM5 microvillar cells in protecting the main olfactory epithelium during excessive chemical exposure. Although generally pleased with the manuscript, the Reviewers felt that the inclusion of some additional experiments that would directly characterize effects of chemical exposure (using either molecular, biochemical, and/or histological assays) on main olfactory epithelium in control and Skn1a-/- mice should be included.

Please provide some basic histological/immunohistological characterization of the olfactory epithelium, or give compelling reasons why this is not possible, with a resubmission target date of approximately 2 months. The full reviews with other suggestions for revision are provided below.

--

Reviewer #1

Advances the Field (Required)

The finding that the TRPM5-expressing microvillous cells in the main olfactory epithelium may help to prevent the epithelium being compromised in its sensory function by excessive chemical exposure is novel and significant.

Comments to the Authors (Required)

In this manuscript, the authors describe electrophysiological and behavioral experiments aiming to address potential functions of a non-neuronal population of microvillous cells, the TRPM5-expressing microvillous cells (TRPM5-MCs), in the main olfactory epithelium (MOE) using Skn-1a knockout mice. TRPM5-MCs were previously shown to respond to various odorous chemicals at high concentration. Skn-1a knockout mice were previously shown to have a morphologically and molecularly normal olfactory sensory neuron population, but lack TRPM5-MCs in their MOE. The authors exposed Skn-1a knockout mice to a mixture of 4 chemicals for 14 days plus chitin powder for 10 minutes each day and then measured odor-evoked responses from the MOE by electroolfactogram and conducted three olfactory-guided behavioral tests. The authors showed that Skn-1a knockout mice had no difference from the control mice, the TRPM5-GFP transgenic mice, in their EOG responses and olfactory-guided behaviors under the standard laboratory housing conditions, but exhibited reduced EOG responses and deficits in these behavioral tests after the chemical-exposure treatment. The chemical-exposure treatment did not significantly alter EOG responses and olfactory-guided behaviors in the control mice. The authors conclude that TRPM5-MCs play a protective role in maintaining the olfactory function of the MOE.

This reviewer finds that the experiments are well designed and well conducted. The chemicals chosen for exposure are justified. The finding that TRPM5-MCs may help to prevent MOE being compromised in its sensory function by excessive chemical exposure is novel and significant. What is obviously missing in this study are the molecular, biochemical, and histological characterizations of the MOE upon the chemical exposure. The future studies in these aspects may help to comprehend the observed physiological and behavioral phenotypes in Skn-1a knockout mice by finding the changes that the chemical exposure exerts on olfactory sensory neurons and on perireceptor events. I have no major concerns about the manuscripts. All my comments are minor.

1. Is Skn-1a expressed in the vomeronasal organ? If yes, how might Skn-1a knockout affect TRPM5-expressing cells in the VNO, the T-maze odor choice test and the block test?

2. In Methods for the buried food test, the authors stated, "any animals that took longer than the cut-off time were excluded from further data analysis". It is better to provide the animal numbers of the total and the excluded mice.

3. In Figure 4E, the lack of difference in the post-exposure test is a key result. It is better to have the N.S. label in the figure.

4. Since the control mice are the TRPM5-GFP transgenic mice, I would suggest using the term "control" to replace the term "wild type (WT)" throughout the paper.

Reviewer #2

Advances the Field (Required)

The functions of TRPM5+ microvillar cells in the main olfactory system are still mysterious. The authors showed that the mice which lack TRPM5+ cells have compromised olfactory functions in a challenged environment, shedding light on the potential role of TRPM5+ cells.

Comments to the Authors (Required)

The authors investigated the potential effects of lack of TRPM5 microvillar cells on the function of the main olfactory system. Using Skn1a knock-out mice, which lack TRPM5 cells, the authors found that after two-week chemical exposure, these mice showed significantly reduced odor and pheromone-evoked EOG responses and compromised olfactory guided food seeking and social odor detection and discrimination behaviors. The experiments were well laid out and the manuscript was clearly written. The manuscript would be further improved by addressing the following concerns.

1. The authors suggest that TRMP5 cells play a protective role for the main olfactory epithelium in the challenge of chemical exposure. It would be helpful to directly demonstrate this point by examining the morphology of the MOE in wild-type and Skn1a-/- mice before and after 2-week chemical exposure.

2. The EOG recordings and the behavioral tests used different sets of odors, which makes the study less coherent. For example, it would be helpful if the authors compare urine induced EOG signals in control and chemical exposed conditions. This is a minor point and the authors should acknowledge this in the Discussion.

3. A few typos. Page 4. Line 60. "Another population MCs expresses" add "of" before MCs. Page 13. Line 263. "statistically difference" should be "different".
